# Roles of the Amygdala and Basal Forebrain in Defense: a Reply to Luyck Et al. and Implications for Defensive Action

**DOI:** 10.1007/s11065-019-09401-y

**Published:** 2019-03-19

**Authors:** Floris Klumpers, Marijn C. W. Kroes

**Affiliations:** 1 Experimental Psychopathology and Treatment Section, Behavioural Science Institute, Radboud University, Nijmegen, The Netherlands; 2 Donders Institute for Brain, Cognition and Behaviour, Radboud University, Nijmegen, The Netherlands; 3 Donders Institute for Brain, Cognition and Behaviour, Radboud University Nijmegen Medical Center, Nijmegen, The Netherlands

**Keywords:** Anxiety, Fear, Amygdala, Bed nucleus of the stria terminalis, Avoidance, Anxiety disorders, Threat, Defensive, Defensive action, Defensive reaction, Threat avoidance, Threat imminence, Nucleus accumbens, Translational

## Abstract

The commentary by Luyck and colleagues on our paper provides many stimulating viewpoints and interpretations of our original study on dissociable responses in the amygdala and bed nucleus of the stria terminalis in threat processing. Here, we reply to some of the points raised and while agreeing with most of the comments also provide some alternative viewpoints. We end by putting forward a research agenda for how to further investigate the roles of these regions in threat processing, with an emphasis on studying their roles in defensive action.

We thank Luyck et al. (2018) for a thoughtful commentary and interesting discussion of the implications of our findings (Klumpers et al., 2017). Their commentary stimulated our thinking, and therefore below we respond to some of the points raised. In addition, we propose further suggestions for how the field might proceed in defining the roles of the amygdala, bed nucleus of the stria terminalis (BNST), and other basal forebrain regions in threat processing.

In our original publication (Klumpers et al., 2017), we reported that neural activity shifted from the BNST to the amygdala when moving from a state of threat anticipation to confrontation with an aversive outcome. Interestingly, only participants who experienced greater childhood maltreatment showed amygdala activation during shock anticipation, without change in BNST activation. Luyck and colleagues rightly caution against over-interpreting the clinical implications of our findings. A critical next step, indeed, for verifying whether the balance between BNST and amygdala activation during threat anticipation contributes to psychopathology is testing a patient population. Regardless, we believe our findings refine the fundamental roles of the BNST and amygdala in threat processing and can inform hypotheses and guide analyses of such future patient studies. Direct comparison between the amygdala and BNST responses to different levels of threat imminence could be particularly informative. Clinical models of stress and anxiety have generally focused on the amygdala, yet many findings also implicate the BNST in anxious psychopathology (Lebow & Chen, 2016). This raises the question whether the amygdala and BNST have a similar contribution to clinical symptoms or if specific aberrations in these regions can lead to unique symptom profiles.

A second point in the commentary considers possible interpretations of those outcomes that appeared discordant in our two large neuroimaging samples. For example, as correctly pointed out by the authors, considerably fewer regions were significantly activated in sample 2 compared to sample 1. Luyck and colleagues link this discrepancy to the difference in temporal unpredictability of the aversive outcome (shocks), which was greater in the second sample. While this is an interesting suggestion, we would like to point out that interpretation of the differences in results between our two samples lacks solid ground without a formal statistical test demonstrating that numerical differences in the mean group response are robust relative to the variance observed within each group. A direct comparison of amygdala and BNST responses to threat anticipation and confrontation with an aversive outcome between our two samples failed to reach significance (all *p*-values > .09). While Luyck and colleagues’ interpretation rests on a solid theoretical framework linking the BNST to temporal unpredictability (Goode & Maren, 2017) and may well be correct, we believe such interpretations of our data may be premature, even regardless of statistical robustness. This is because the two samples differed in a wide range of characteristics other than temporal unpredictability. Most notably, the experiment for the first sample was (I) shorter and contained fewer trials, (II) was scanned at a lower temporal resolution at an MR scanner from a different manufacturer (Philips vs Siemens) and (III) the samples were different that the first sample contained fewer subjects (70 vs 108; leading to differences in statistical power) and consisted only of males (vs. 70% females in sample 2). While these factors were not of direct interest to our study, they all potentially contributed to differences in results between samples. Therefore, while the converging findings *across* our two independent samples were robust regardless of these different experimental parameters and therefore more convincing, interpretations regarding differences in results *between* our samples rest on limited evidentiary value.

Luyck et al. also highlight that our results are not a one-to-one match with models derived from years of rodent literature, where the amygdala has been posited as important for particularly post-encounter phases and the BNST might be involved in pre-encounter phases where threat is uncertain. We fully agree and particularly support their suggestion for an important distinction between human and rodent work in terms of the level of threat. For ethical reasons, threat levels in human studies are limited compared to rodent studies. We propose that experimental conditions of threat anticipation in humans may thus be shifted down on the threat imminence continuum compared to similar studies in rodents. This might explain the inconsistent amygdala activity in humans when threat is distant while observing clear activation of the BNST, even with short cue durations (Klumpers et al., 2017; Fullana et al., 2016; Mechias et al., 2010; Fox & Shackman, 2019).

We would like to add an additional explanation for the apparent discrepancy in amygdala and BNST functioning between our findings in humans and those in the rodent literature. One proposed (core) function of emotions is to invigorate the propensity of appropriate behavioral responses (Frijda, 1986; Barrett, 2017). When threat appears at a distance, animals may reduce foraging behaviors and try to stay clear of danger. At the same time the inherent uncertainty about threat can also create an urge to explore and further assess risk. The latter requires staying in contact with the potential threat (e.g., visually) and sometimes even approaching it. When threat comes near, animals may stop all motion, and passively or actively try to avoid threat. At close confrontation with a threat, animals may flee or fight. The exact appropriate behavioral responses will thus dependent on the imminence of threat but also on the specific species confronted with threat. Many species-specific responses along the threat imminence continuum are reactions (e.g. running, jumping, flying, burrowing), which may require distinct neural mechanisms to deal with the threat. We, however, still know little about these species-specific threat-reactions and the differences in the underlying neurocircuitry. Most threat studies in rodents are conducted in small test environments limiting the behaviors animals will express. Perhaps even more problematic, human participants in threat studies are generally instructed to refrain from any movements while constantly looking at the screen to optimize psychophysiological recording (e.g. startle electromyography, galvanic skin conductance responses and fMRI). Restricting behavioral responses in threat experiments thus provides a limited assay of threat behaviour (Gentry et al., 2016; Hamel et al., 2017; LeDoux et al., 2017; Beckers et al., 2013; Cain, 2019) and as a result instrumental threat-related behaviors in rodents and humans remain relatively understudied (LeDoux et al., 2017) notwithstanding recent progress (Aupperle et al., 2015; Schlund et al., 2016; Boeke et al., 2017; Moscarello & LeDoux, 2013; Ramirez et al., 2015). In our study we also did not probe defensive reactions such as approach and avoid tendencies. An additional explanation for differences between our findings and those of studies in rodents may thus be the degree to which similar and distinct neural mechanisms for threat reactions are invigorated. Increased understanding of species-specific threat reactions could thus help to explain differences in observations between threat studies in rodents and humans.

Philosophers, psychologists and behavioral neuroscientists alike regard conflict between approach and avoidance motivational tendencies as central to anxiety (Kierkegaard, 1980; Blanchard et al., 2011; McNaughton & Corr, 2004; Corr, 2013; Kirlic et al., 2017). Understanding the neural mechanisms of instrumental threat-related approach and avoidance behaviors and their conflict is therefore critical to understand neural circuits driving anxiety and especially pathological anxiety because excessive avoidance is a hallmark symptom in stress- and anxiety disorders (Craske & Stein, 2016). An interesting perspective for future research is, therefore, to investigate potentially distinct roles of the amygdala and BNST in threat-actions. Current neurobiological models of avoidance are based largely on threat avoidance learning experiments with rodents and highlight the importance of amygdala connections to the ventral striatum, particularly the nucleus accumbens. Human and rodent decision-making paradigms have identified a similar pathway underlying instrumental approach reactions and the ventral striatum has long been implicated in appetitive processing (Fig. 1). Distinct amygdala-nucleus accumbens pathways thus appear involved in both approach and avoidance reactions of rewarding and aversive outcomes. The nucleus accumbens borders the BNST in both rodents and primates and the regions have tight interconnections. Evidence implicates the BNST in avoidance (Duque-Wilckens et al., 2018) consistent with its strong connections to striatal and motor regions (Klumpers et al., 2017). Indeed functionally defined sub-regions of the BNST have recently been implicated in driving oppositely-valenced approach and avoid reactions via their connections to the hypothalamus (Giardino et al., 2018). However, to what extent the BNST’s role may be distinct from the amygdala is unclear, potentially because most threat-avoidance studies take place on the proximal end of the threat imminence continuum (Cain, 2019). We thus call for additional research on the role of the BNST and amygdala in driving behavioral reactions along the entire threat imminence continuum.

**Fig. 1:**
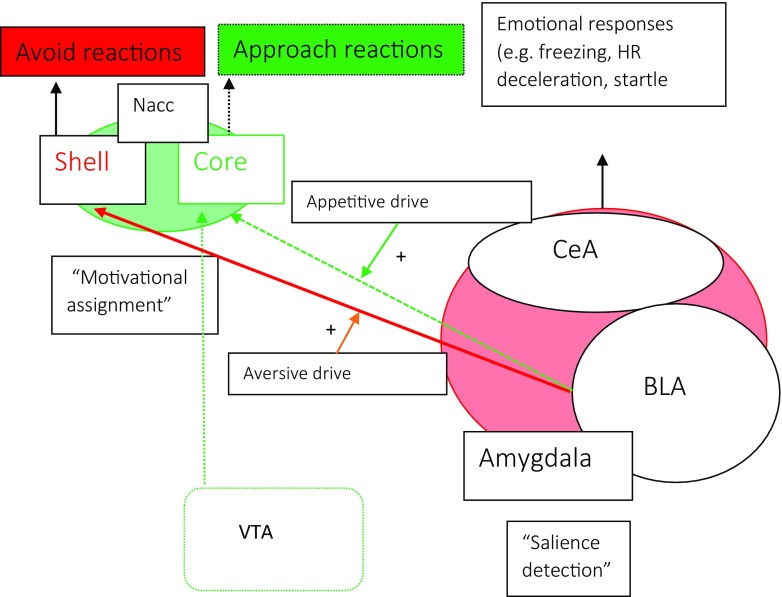
A neural model for the integration of threat and reward in costly avoidance decision making. The Amygdala and NAcc each have a role in driving both approach and avoid behavior (Gentry et al., 2016; Hamel et al., 2017; Schlund & Cataldo, 2010). However, evidence from (pharmacological) lesion and stimulation studies in rodents indicates a relative importance for the BLA in driving avoid responses (Choi & Kim, 2010; Duque-Wilckens et al., 2018; Burgos-Robles et al., 2017; Terburg et al., 2018) and a relative importance for the NAcc (core) in approach responses (Hamel et al., 2017; Nachev et al., 2015). One explanation is that in absence of BLA input reward-related inputs from the ventral tegmental area (VTA) continue to drive approach via the NAcc (Cisneros-Franco & de Villers-Sidani, 2018). This model, however, is still sorely lacking a role for the BNST, which has strong interconnections to both the amygdala and NAcc

Finally, as described above, conflicting motivational tendencies might be central to anxiety and therefore it will be highly informative for a better understanding of clinically relevant mechanisms to include conditions of conflicting motivational tendencies in research on defensive-reactions. As opposed to the majority of previous work on defensive reactions, daily life rarely involves situations with unambiguously optimal behavioral reactions and including these conflicting motivational conditions in threat studies would thus allow more accurate modelling of pathological avoidance (Pittig et al., 2018; Krypotos et al., 2018). Illustrating this point, threat avoidance per se is not pathological and might even be a healthy and efficient strategy (Boeke et al., 2017). Pathological anxiety, however, is characterized by avoidance under ambiguous conditions that is persistent even in the face of high costs such as social isolation and functional impairment (Pittig et al., 2018). Thus, studies investigating the role of the amygdala and BNST using paradigms that allow more active behaviors and include conditions of approach/avoidance conflict are highly anticipated.
